# Implant-based immediate reconstruction in prophylactic mastectomy: is the caudal dermis flap a reliable alternative to synthetic mesh or acellular dermal matrix?

**DOI:** 10.1007/s00404-021-06244-y

**Published:** 2021-09-23

**Authors:** N. Heine, V. Hoesl, S. Seitz, L. Prantl, V. Brebant

**Affiliations:** 1 Department of Plastic Surgery, University Hospital Regensburg, Regensburg, Germany; 2 Department of Gynecology, Caritas Hospital St. Josef and University Hospital Regensburg, Regensburg, Germany

**Keywords:** Caudal dermis flap, mastectomy, breast reconstruction, macromastia, ptosis

## Abstract

**Introduction:**

The demand for prophylactic mastectomy has increased significantly over the last 10 years. This can be explained by a substantial gain of knowledge about the clinical risk and outcome of patients with high risk mutations such as BRCA1 and 2, the improved diagnostic possibilities for detecting the genetic predisposition for the development of breast cancer and the awareness for those mutations by health care professionals as well as patients. In addition to expander-to-implant reconstruction and microsurgical flap surgery, definitive immediate reconstruction with subpectoral insertion of breast implants is often preferred. The prosthesis is covered at its inferior pole by a synthetic mesh or acellular dermal matrix. In these cases, in addition to the silicone prosthesis, a further foreign body must be implanted. This can be exposed in the event of wound healing disorder or necrosis of the usually thin soft tissue covering after subcutaneous mastectomy, thus calling into question the reconstructive result. In this study, the coverage of the lower pole by a caudal deepithelialized dermis flap, which allows the implant to be completely covered with well vascularized tissue, is compared to coverage by a synthetic mesh or acellular dermal matrix.

**Patients and methods:**

From January 2014 to June 2020, 74 patients (106 breasts) underwent breast reconstruction following uni or bilateral prophylactic mastectomy. Reconstruction was performed with autologous tissue (15 breasts), with tissue expander or implant without implant support (15 breasts), with implant and use of an acellular dermal matrix or synthetic mesh (39 breasts) and with implant and caudal dermis flap (37 breasts).

In this study, we compared the patients with implant and dermal matrix/mesh to the group reconstructed with implant and dermal flap.

**Results:**

In the group with the caudal dermis flap, 4 patients developed skin necrosis, which all healed conservatively due to the sufficient blood supply through the dermis flap. In the group with the use of a synthetic mesh or acellular dermal matrix, skin necrosis was found in three cases. In one of these patients the implant was exposed and had to be removed.

**Discussion:**

For patients with excess skin or macromastia, the caudal dermis flap is a reliable and less expensive option for complete coverage of an implant after prophylactic mastectomy. In particular, the vascularized dermis flap can protect the implant from the consequences of skin necrosis after prophylactic mastectomy.

## Introduction

Due to a substantial gain of knowledge about the clinical risk and outcome of patients with high risk mutations such as BRCA1 and 2, improved diagnostic possibilities for detecting the genetic predisposition for the development of breast cancer and the awareness for those mutations by health care professionals as well as patients, the demand for prophylactic mastectomy has increased significantly over the last 10 years. Many patients with breast cancer in their medical history ask for a genetic analysis. Gene mutations, for example in the BRCA1/BRCA2 gene, are frequently detected in this context [18]. Similarly, patients without a personal history of cancer but with a high familial risk of developing breast or ovarian cancer can be identified after investigations of mutation carriers in the family environment.

Even women without genetic modification increasingly want prophylactic mastectomy of the contralateral breast after breast cancer, although close follow-up in these women is equivalent to surgery in terms of long-term survival. As long as no additional potential risk of disease is seen in their familial environment, these patients are not encouraged to undergo contralateral prophylactic mastectomy in accordance with the Contralateral Prophylactic Mastectomy (CPM) Consensus Statement of the American Society of Breast Surgeons [2,9].

The mean age of onset to develop breast cancer in women is in their sixth and seventh decade of life. Patients who consider a prophylactic mastectomy are on average much younger and more body-conscious, so that typically very high demands are placed on the reconstruction of the breast [11,14,17,20].

In addition to the classic procedures with a two-stage expander/implant reconstruction and autologous flaps, these patients often prefer immediate one-stage reconstruction with a definitive anatomical implant. Synthetic meshes or acellular dermal matrices (ADM) are usually inserted to cover the subpectoral placed implant inferiorly [3,5,6,12,16]. In these cases, a further foreign body must be inserted in addition to the silicone prosthesis with the relevant risk of exposure in case of skin necrosis after subcutaneous mastectomy, potentially followed by failure of the complete reconstruction [1].

An alternative in patients with mild to severe ptosis mammae or macromastia is the caudal dermis flap, which draws its blood supply from the subdermal vessels and is sutured to the caudal edge of the mobilized pectoralis major muscle [4]^7^. This ensures complete coverage of the implant with well-vascularized autologous tissue even before skin closure.

The study was approved and given informed consent by the local ethics committee.

## Patients and methods

Between January 1, 2014 and March 31, 2020, 74 patients with a total of 106 breasts underwent prophylactic mastectomy unilaterally or bilaterally at the University Breast Cancer Center Regensburg.

The following techniques have been used for reconstruction:

-Autologous DIEAP flap (uni-/ or bilateral Deep Inferior Epigastric Artery Perforator flap).
-Expander/Implant without additional support.
-Implant-based reconstruction with additional mesh/ADM.
-Implant-based reconstruction with the caudal dermis flap.

All patients were operated on by a team of a plastic surgeon and an oncological gynecologist.

### Indication

23 patients of the collective were carriers of a BRCA1 mutation, 26 patients were carriers of a BRCA2 mutation. One patient each had Li-Fraumeni syndrome (TP53 mutation), CHEK2 mutation or ATM mutation. These mutations were regarded as pathogenic. In 3 women there was a familial accumulation of breast cancer without genetic mutation.

In 32 patients a bilateral prophylactic mastectomy was performed. 42 patients already had breast cancer in their history, so that a prophylactic mastectomy was performed contralateral to the breast cancer (Table 1).

**Table 1:** Indications for prophylactic mastectomy

Indication	Number of patients	Number of cases (breasts)
BRCA1 mutation	23	33
BRCA2 mutation	26	46
TP53 mutation	1	1
CHEK2 mutation	1	1
ATM mutation	1	1
Familial accumulation	3	5
Others	19	19
Contralateral breast cancer (unilateral reconstruction)	42	42

Twelve patients (15 breasts) received a breast reconstruction with an autologous DIEAP flap.

In 62 patients (91 breasts) an implant-based reconstruction was performed. A single-stage implant-based, submuscular immediate reconstruction after subcutaneous mastectomy was selected in 6 patients (7 breasts). In 7 patients (8 breasts) a two-stage procedure with expander insertion and change to an anatomical implant was performed 6 months later. In these two procedures, none of the 13 patients (15 breasts) received a caudal implant cover.

In 25 patients (39 breasts), caudal coverage of the subpectoral implant was performed as part of the immediate reconstruction after subcutaneous mastectomy with a synthetic mesh or acellular dermal matrix (ADM). In total, a SERI mesh (Sofregen Medical Inc., Boston/USA) was used in 2 patients (4 breasts). In 3 patients (5 breasts) a Vicryl mesh (Ethicon, USA) was inserted. In 20 patients (30 breasts) a bovine dermal matrix was used. Out of these, one patient (1 breast) received a Surgimend matrix (Polytech, Germany), 2 patients (4 breasts) Strattice and 17 patients (25 breasts) Artia (both LifeCell Corporation, Branchburg, New Jersey, USA).

In 24 patients (37 breasts), a vascularized caudal dermis flap was used to cover the lower pole of an anatomical implant during immediate reconstruction (Table 2).

**Table 2:** Techniques of reconstruction

Technique of reconstruction	Number of patients	Number of cases (breasts)
Autologous tissue (DIEAP)	12	15
Implant only	6	7
Expander only	7	8
Implant with synthetic mesh/ADM	25	39
Implant with caudal dermis flap	24	37

In this study, only the implant-based reconstructions with caudal support by mesh/ADM or dermis flap were included. The aim of the study was to evaluate the safety and efficacy of the caudal dermis flap to cover the inferior pole of the implant compared to an implant-based reconstruction with coverage of the caudal pole by non-vascularized tissue (synthetic mesh or ADM).

### Surgical technique of the caudal dermis flap

**Fig. 1:**
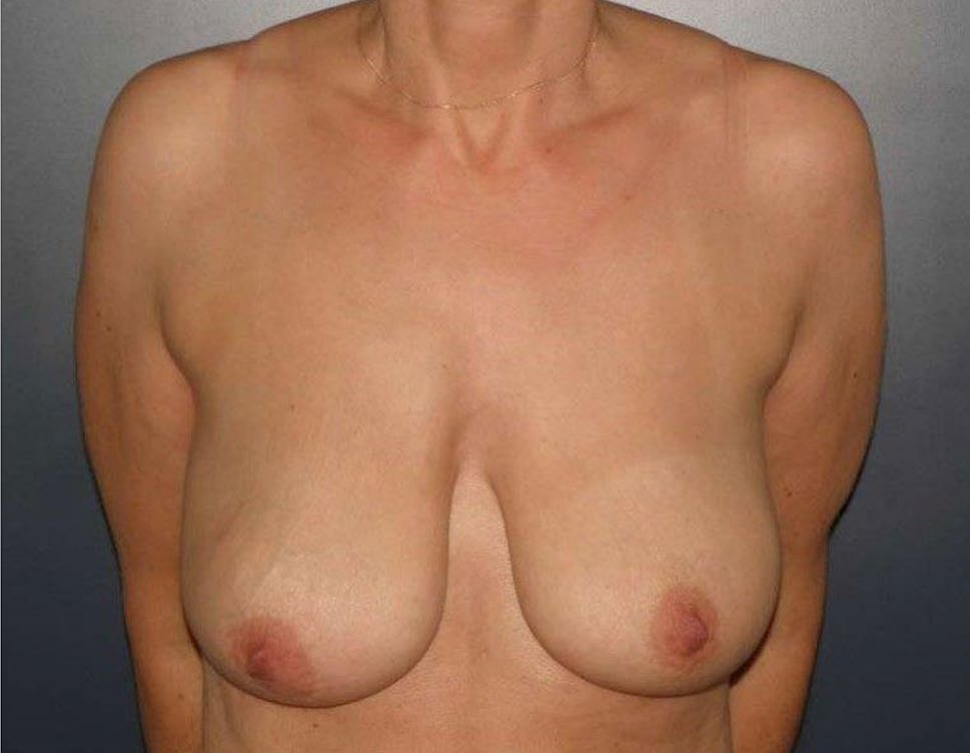
Patient with ptosis grade 2 (BMI 21)

**Fig. 2:**
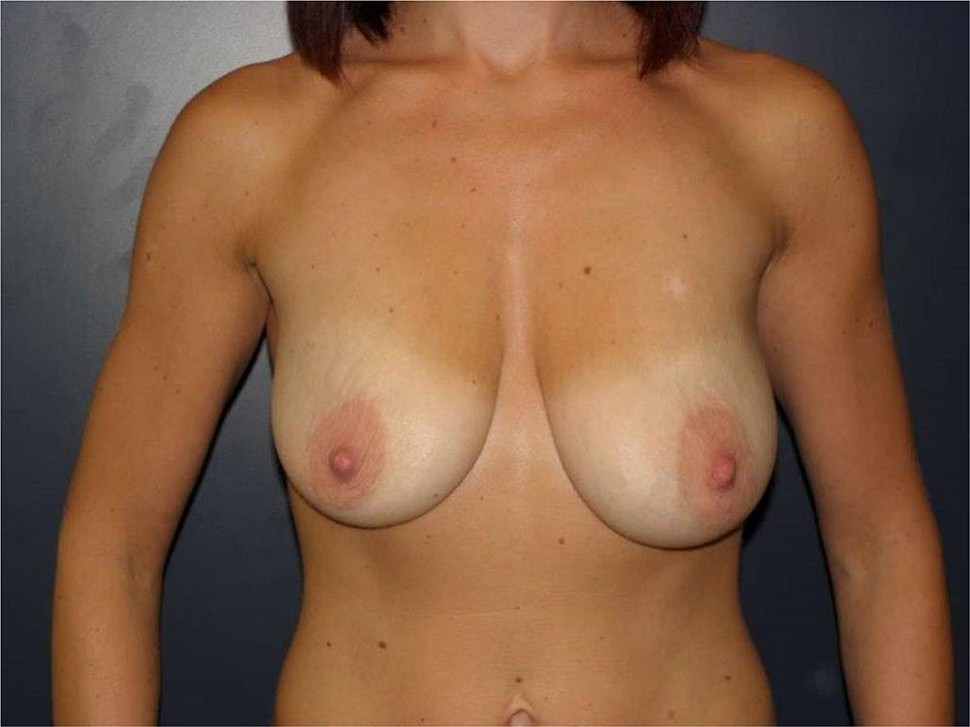
Patient with ptosis grade 2 and asymmetry (BMI 18)

Inclusion criteria for the technique have been patients with breast ptosis or macromastia, a nipple-IMF distance of 11 cm or more and a sternal-notch-to-nipple distance (SNND) of at least 25 cm (Figs. 1, 2).

A T-scar reduction figure was designed as a surgical access for prophylactic mastectomy, depending on the expected implant size. The caudal part of the skin was deepithelialized according to the Wise pattern [9]. Leaving an adipodermal flap of 5 mm of thickness in average with preservation of the subdermal vascular plexus, the gland was then removed from the fascia.

In 17 patients (28 breasts) the nipple-areola complex (NAC) was preserved on a medial pedicle. For natural contouring of the lower breast, the dermis was incised medially and laterally of the flap for about 2–3 cm to allow a tension-free connection to the lower edge of the pectoralis muscle. Subcutaneous mastectomy was then performed, leaving the epipectoral fascia untouched.

**Fig. 3:**
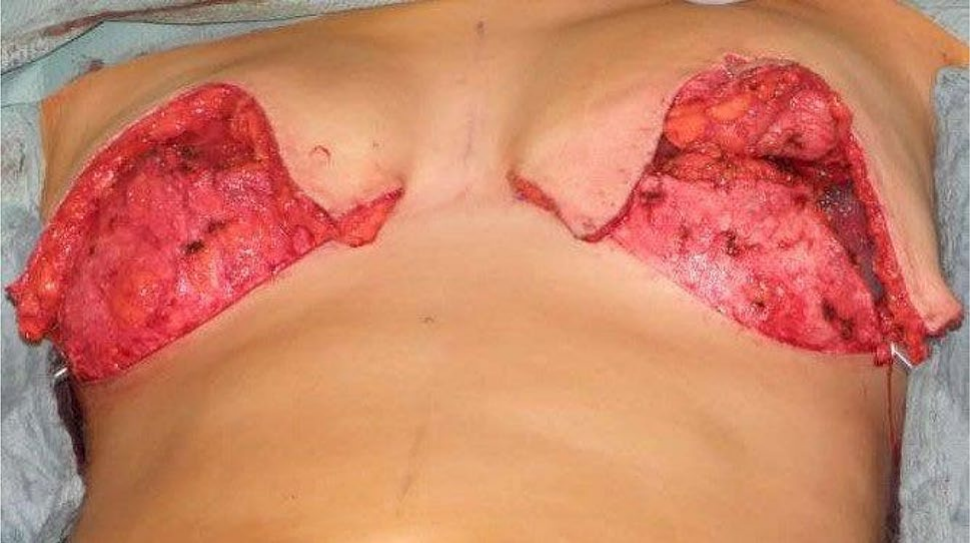
Bilateral caudal dermis flap intraoperatively

The major pectoralis muscle was then lifted from its laterocaudal margin and a subpectoral pocket was prepared. After subpectoral insertion of an appropriate anatomical implant, the caudal dermis flap with its free edge was sutured to the lower edge of the muscle. The asymmetrical shape of the dermis flap, which follows the shape of the "Wise pattern" with a larger portion laterally, corresponds to the deficit in coverage remaining caudolateral to the pectoralis muscle. Laterally, the serratus fascia was mobilized to the anterior axillary fold to complete the coverage of the implant by suturing it to the margin of the lateral pocket (Fig. 3).

The skin flaps were now placed over the vascularized pocket and closed in two layers after insertion of a suction drainage. If the NAC had not been removed, it was rotated cranially on its medial pedicle, placed and sutured to the point of highest projection of the reconstructed breast.

To visualize the blood supply, an examination of the vascularization with fluorescence imaging by administration of indocyanine green (IC-View, Novadaq/Stryker, Canada) was carried out in 19 operations after closure of the implant pocket, but before closure of the skin envelope. A lack of the blood supply at the skin envelope could be visualized at the same time and, if necessary, cured by local resection (DeVita R).

## Results

In 74 patients who underwent prophylactic mastectomy between January 2014 and March 2020, a total of 106 breasts were removed. Patients with cancer on the ipsilateral side of the breast in their own medical history were not included in this study.

32 women had no history of breast cancer. In these cases, a bilateral prophylactic mastectomy was performed (64 breasts). In 42 patients the contralateral side was treated for breast cancer (42 breasts).

The average BMI of all patients was 24 (18–33). The mean age was 41.6 years (22–64).

The minimum follow-up period was 6 months.

For this study, 49 patients (76 breasts) with implant-based reconstruction and caudal support were enrolled.

Based on the technique used, the patients were divided into two groups:

- Group A (submuscular implant with synthetic mesh or ADM)
- Group B (subpectoral implant with caudal dermis flap)

Group A included 25 patients (39 breasts) who received a caudal covering of the implant with an artificial mesh or ADM after subpectoral implant placement. The mean age of these patients was 38 years (24–58 years) and their BMI was 22.4 (19–27).

The nipple-areola complex was preserved in 19 patients (33 breasts).

An anatomical implant from Mentor was placed in six reconstructions. Reconstruction with a Polytech anatomical implant was performed 33 times. The volume of the implants varied between 210 and 610 cc, with an average of 380 cc.

Eleven patients had a history of contralateral breast cancer. Two of these patients had undergone contralateral radiotherapy and seven had undergone chemotherapy. None of these pretreated women experienced postoperative complications.

Five patients of this group (8 breasts; 20.5%) were smokers. In one of them a bilateral seroma was detected and treated (5.1%).

Three breasts had skin necrosis or deep wound healing disorder as complications (7.7%). As a result, in one patient the Vicryl net was exposed with consecutive implant loss (2.6%). The other two cases showed conservative healing of skin necrosis/wound healing disorder.

Severe scar deformation was observed in one patient as a minor complication (2.6%). One woman received a blood transfusion due to postoperative bleeding (2.6%).

**Fig. 4:**
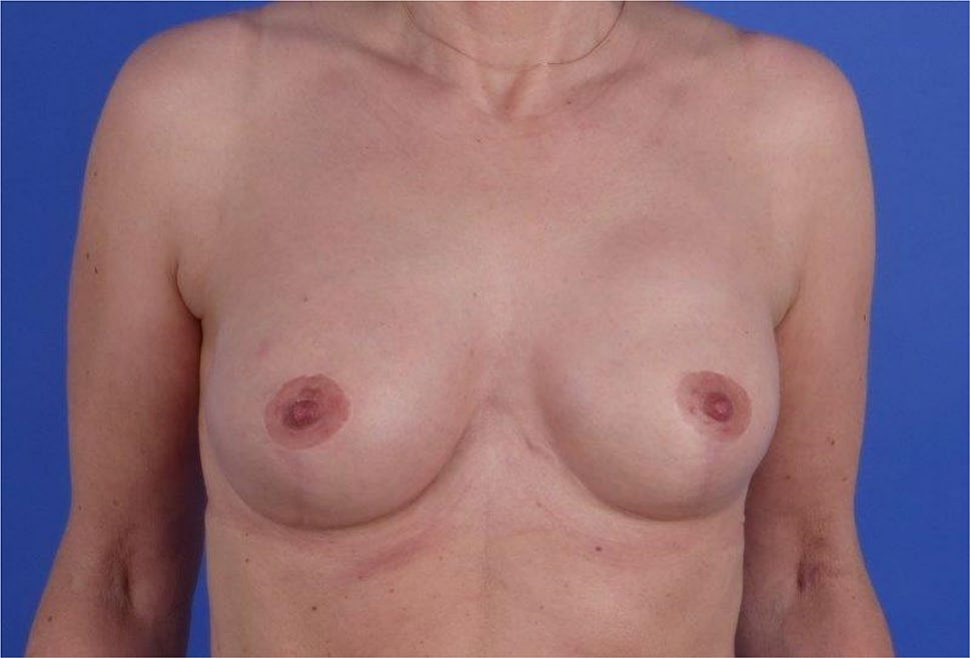
Bilateral caudal dermis flap postoperatively (anatomical implants, 305 cc, NAC preserved)

**Fig. 5:**
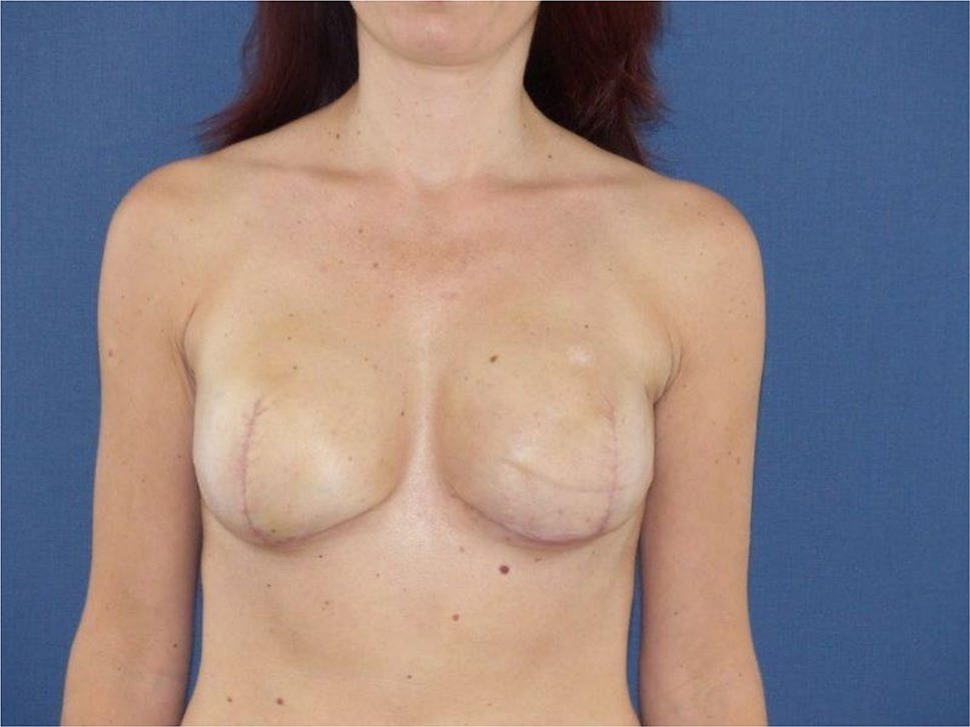
Bilateral caudal dermis flap postoperatively (anatomical implants, 320 cc, NAC removed)

Group B included 24 patients (37 breasts) with ptosis or macromastia. Thirteen patients in this group received bilateral and eleven unilateral reconstructions. In all 37 reconstructions, complete coverage of the implant was achieved by a caudal dermis flap, forming a closed, vascularized pocket (Figs. 4, 5). These patients were 43 years old on average (22–64). The BMI was 24.4 in average (19–33).

The nipple-areola complex was preserved in 17 patients (28 breasts).

An anatomical implant from Mentor (Mentor LCC, Irvine, CA, USA) was selected for reconstruction in 14 breasts. An anatomical implant from Polytech (Polytech Health & Aesthetics, Dieburg/Germany) was used for 23 breasts. The implant volume ranged from 210 to 570 cc (average 400 cc).

Eleven patients in group B had a history of breast cancer. Ten women had received chemotherapy and five radiotherapy of the contralateral breast. None of these patients had any complications.

Three patients were smokers (4 breasts, 10.8%). One of these patients developed skin necrosis.

The reconstruction did not fail in any case. A total of four breasts (10.8%) developed skin necrosis. These women healed conservatively in all cases.

In two patients a hematoma occurred postoperatively (5.4%). One of these hematomas required surgical revision (2.7%).

Looking at the whole collective in which a prophylactic mastectomy was performed, skin necrosis occurred in 12 breasts (11.3%). Within the two groups compared in the study, three cases of skin necrosis occurred after insertion of a synthetic mesh /ADM and four after use of the caudal dermis flap.

While all reconstructions with covering of the implant by caudal dermis flap were successful, implant reconstruction using a Vicryl mesh failed (implant loss after mesh exposure).

A total of eight patients (12 breasts) of the entire collective were smokers (11.3%). One of these patients developed skin necrosis after breast reconstruction with implant and a caudal dermis flap (8.3%). This could be healed conservatively. Smoking was therefore not considered a relevant risk factor for skin lesions.

Seven patients of group A (28%) and ten women of group B (42%) hat received chemotherapy; none of them suffered skin lesions (Table 3).

**Table 3:** Complications after prophylactic mastectomy

Technique of reconstruction	Cases of skin necrosis/ wound healing disorder (breasts)	Cases of reconstruction failure (breasts)
Implant with synthetic mesh/ADM	3	1 (implant loss)
Implant with caudal dermis flap	4	0

## Discussion

The goal of risk-reducing mastectomy is to remove healthy glandular tissue facing an increased risk of developing breast cancer. When planning a prophylactic operation, no consideration must be given to a current tumor disease, so that a skin-sparing method is justified. The oncological safety of skin-saving procedures has been confirmed in many studies as well as by statements of the American Society of Breast Surgeons [13].

The preservation of the original skin envelope with simultaneous removal of the mammary gland in mostly younger, often slim patients poses a special challenge for the blood circulation of the remaining skin. In our study, 10.8% of the women with a caudal dermis flap suffered skin lesions or necrosis, which is quite similar to other series. Demiri [8] published results from fifty patients with 65 operated breasts and reconstruction with a caudal dermal flap, 16.9% of whom had skin lesions.

Gianotti et al. [10] were able to show that after mastectomy, with an average thickness of the remaining skin envelope of 5.5 mm, glandular tissue still remains on the skin in 29.9% of operations. To remove the gland almost completely means a very intensive thinning of the skin envelope, accepting a considerable risk of affecting the blood supply to the remaining skin.

Inbal et al. [11] could demonstrate that, according to the technique with the caudal dermis flap in addition to implant-based reconstruction, resected breast weight above 700 g was associated with major complications significantly. In our study, only patients with prophylactic mastectomy and a resected breast weight lower than 700 g were compared.

Potter et al. [15] had regarded 18% of revisions and 9% implant loss in a prospective multicenter study with 2655 implant-based breast reconstructions.

Any necrosis of the skin carries the risk of exposure of the foreign material, with potential failure of the reconstruction. The use of autologous tissue, especially for bilateral reconstruction, is often limited in the mentioned target group of patients with the demand for prophylactic surgery, so that from all prophylactic operations in our series, 86% of the interventions were performed implant-based.

While the mean BMI of the patients with autologous reconstruction after prophylactic mastectomy in our series was 28.3, patients with an implant-based reconstruction had a BMI of 23.3, so that the decision for the surgical procedure was also influenced by the patient`s weight and available donor regions.

For Vlajcic et al. [19], the caudal dermis flap reacts not only as a coverage of the lower pole but also as suspension preventing laterocaudal sagging of the implant.

In patients with ptosis mammae or macromastia, the technique of the caudal dermis flap allows the implant to be placed in a completely vascularized pocket, before the skin envelope is closed [11]. The advantage of the caudal coverage of the implant with the dermis flap lays -next to the avoidance of an additional foreign body- in the protective effect of well-vascularized tissue before closing the skin envelope.

Our study demonstrates that skin necrosis, which potentially can lead to exposure of the deep layer and loss of reconstruction when using synthetic mesh or ADM, can heal conservatively due to adequate vascularization of the dermis flap. As a result, even in patients who are not eligible for a complete autologous tissue reconstruction, the caudal dermis flap achieves a higher safety to preserve the implant after skin necrosis by coverage of the implant with well-vascularized tissue.

In opposite to other studies [8], the overall complication rate was low in our series. Only one implant loss was registered following skin necrosis after insertion of a synthetic mesh.

Friedman et al. [9] published a technique, where the caudal dermis flap was used in combination with ADM. As a finding, the failure rate was significantly higher and the technical and economical effort was rising, so we don`t recommend the use of the dermis flap in combination with synthetic mesh or ADM.

Limitations of our study are the limited number of cases (39 vs. 37 breasts for the two compared groups) and the different inclusion criteria for the two groups (skin excess necessary for group B with the caudal dermis flap).

## Conclusion

In our study of prophylactic mastectomy, we could demonstrate that the vascularized caudal dermis flap is a reliable and safe alternative to cover the lower pole of a subpectoral placed implant for patients with sufficient skin excess in case of macromasty or ptosis mammae.
